# Seeking care for epilepsy and its impacts on households in a rural district in southern Malawi

**DOI:** 10.4102/ajod.v2i1.54

**Published:** 2013-09-30

**Authors:** Alister Munthali, Stine H. Braathen, Lisbet Grut, Yusman Kamaleri, Benedicte Ingstad

**Affiliations:** 1 Centre for Social Research, University of Malawi, Malawi; 2 SINTEF Technology and Society, Oslo, Norway; 3 Department of Community Medicine, University of Oslo, Norway

## Abstract

**Background:**

Epilepsy is a disability as defined in the 2012 Disability Act of the Government of Malawi.

**Objectives:**

This article explores the health-seeking behaviour of people with epilepsy in a rural town in southern Malawi and how having a person with epilepsy impacts on the households’ productivity.

**Method:**

A snowball approach was used to identify persons with various forms of disabilities. The article is based on a bigger study carried out in Malawi which explored how persons with disabilities seek health care. In this bigger study, a total of 63 interviews were done with persons with disabilities or their guardians. Eight of the 63 interviews were with persons with epilepsy and this article is based on these interviews.

**Results:**

The study found that persons with epilepsy seek both traditional and modern medicines to treat the condition. Informants mentioned that barriers to accessing western treatment include lack of medicines, congestion at health facilities, lack of knowledge about epilepsy, misdiagnosis by health workers and the belief that epilepsy caused by witchcraft cannot be treated by western medicine. The study also highlights the wider impacts of epilepsy on the household such as the failure of children to attend school, children dropping out of school, stigma and discrimination and households being driven deeper into poverty as a result of seeking care for members with epilepsy.

**Conclusion:**

The existing barriers to accessing treatment for epilepsy can be addressed by using a combination of public education, simple treatments and regular reviews. Ensuring constant availability of drugs for the treatment of epilepsy is key to effective treatment of the condition. This would contribute to closing the treatment gap for epilepsy as advocated by the Global Campaign against Epilepsy.

## Introduction

Epilepsy refers to disorder of the brain characterised by recurrence of unpredictable interruptions of the normal brain function called epileptic seizures (Fisher *et al*. 2005; World Health Organization [WHO] 2012). An individual has a 1 in 10 chance of experiencing at least one epileptic seizure in his or her life. Active epilepsy has been defined as one that has caused two or more unprovoked seizures on different days in the year prior to the assessment date (WHO 2004).

Biomedically, there are two broad categories of epilepsy: *symptomatic epilepsy* resulting from particular identifiable causes such as birth asphyxia, head injury and meningitis, whilst *idiopathic epilepsy* may develop without any identifiable cause, of which the WHO (2012) says that such a type of epilepsy has an underlying genetic basis. Whilst this classification has been accepted all along, the International League against Epilepsy Commission on Classification and Terminology revised the concepts, terminology and approaches for classifying seizures and forms of epilepsy based on aetiology. The recommendation is that the following classification should be used, namely, (1) genetic epilepsy, which is a direct result of a genetic cause; (2) structural-metabolic epilepsy, which results from a separate structural or metabolic condition; and (3) unknown, which implies that the cause of epilepsy is unknown and there is a need for further research (Berg & Scheffer 2011).

In African societies the causes of epilepsy include childhood febrile convulsions, various infections, injuries, tumours and vascular diseases (Diop *et al*. 2003). Asindi *et al*. (1995) found that birth asphyxia, infections and hypoglycaemia were causes of epilepsy amongst infants in 48% of the cases. Epilepsy is the most common serious chronic brain disorder estimated to affect at least 50 million people in the world, of which 10 million live in Africa alone (Diop *et al*. 2003). The prevalence of active epilepsy in developing countries ranges from 5 to 10 per 1000 people (Scott, Lhatoo & Sander 2001). Over the years, there have been great advances in the diagnosis and treatment of epilepsy, but to date 8 million people with epilepsy are not treated (Diop *et al*. 2003; WHO 2004) due to poor infrastructure, insufficient availability of drugs and shortage of human resources (WHO 2004; Diop *et al*. 2003), amongst other factors. In order to close the prevailing treatment gap, in 1997 a Global Campaign against Epilepsy was jointly launched by the WHO, the International League Against Epilepsy and the International Bureau for Epilepsy (Scott *et al*. 2001; WHO 2004).

### Background about Malawi

Malawi is a small country located in Central Africa and has a population of 13 million people (National Statistical Office 2008). There are 24 districts and Lilongwe is the capital city. A study conducted by the Federation of Disability Organisations in Malawi found that 2.8% of the population in Malawi constitutes persons with epilepsy (Amos & Wapling 2010), and this is lower than an earlier estimate of 5.2% by the WHO (2004).

Whilst treatment for epilepsy is available, access still remains a major challenge in Malawi (Amos & Wapling 2010). Whiteley (2005) says that seizures are often complicated by burns when people fall onto fires or stoves. Of 100 Malawian adults admitted in a hospital with burns, 36 had sustained these during an epileptic seizure (Buchanan 1972, cited in Watts 1989). This demonstrates that epilepsy is one of the major non-communicable diseases in Malawi, hence should be given priority. The Ministry of Health provides treatment for persons with epilepsy and the condition is amongst the diseases prioritised in the Essential Health Package (EHP) in the 2011–2016 Malawi Health Sector Strategic Plan (Ministry of Health 2011).

It has been demonstrated that conventional anti-epileptic drugs such as phenobarbitone can be used successfully in Malawi (Watts 1989) and that 2.8% of Malawians suffer from this condition. Epilepsy and other neurological diseases have not received much attention in terms of increasing patients’ access to appropriate drugs. Whilst some studies on epilepsy have been done (e.g. Amos & Wapling 2010; Chilopora *et al*. 2001; Watts 1989; Whiteley 2005), there is limited information on health seeking behaviour for people with epilepsy and how having a person with epilepsy within the household impacts on the household welfare and productivity.

### About the study

This article is based on a bigger study conducted by SINTEF, the Foundation for Scientific and Industrial Research, a research organisation based in Oslo, Norway, together with the Faculty of Medicine at the University of Oslo in Norway; and the Centre for Social Research of the University of Malawi. The major objective of the study was to explore access to health services by persons with disabilities. It was based on the premise that whilst international conventions such as the United Nations Convention on the Rights of Persons with Disabilities call for equality in accessing health care for all people including persons with disabilities, the situation at national level in most countries is different; people with disabilities are often neglected in the provision of social services (Eide & Loeb 2006).

A few other studies have been done exploring access to health care by people with disabilities in Malawi. For example in 2003 a survey of living conditions amongst people with disabilities in Malawi was conducted, using the International Classification of Functioning, Disability and Health (ICF) Model to identify persons with disability. The model defines disability in terms of participation restriction and activity limitations (WHO 2005). This study showed that whilst 84.2% of the people with disabilities were aware of health services and 83.4% needed services, only 61% actually received the services (Eide & Loeb 2006). These results demonstrate that even though services may be available and the Constitution of the Republic of Malawi calls for provision of services to all Malawians (Government of Malawi 1994), people with disabilities have problems accessing these services mainly because of their disability.

The survey on living conditions does not really explain the health seeking behaviour of people with disabilities. The current study was therefore conceptualised to produce some empirical evidence on the health-seeking behaviour of people with specific disabilities and their families, and how the presence of people with specific forms of disability impacts on the welfare and productivity of the household. Whilst people with various forms of disabilities were interviewed in the overall study, this article focuses on the health-seeking behaviour of persons with epilepsy and how this impacts on households’ welfare and productivity. The focus on epilepsy is important for Malawi as the condition has not received much attention over the years.

In Malawi, as well as internationally, epilepsy is considered as a disability. The *Malawi Disability Act 2012* defines disability as follows:

‘Disability’ means a long-term physical, mental, intellectual or sensory impairment which, in interaction with various barriers, may hinder the full and effective participation in society of a person on equal basis with other persons. (Government of Malawi 2012:6)

Since epilepsy is a neurological disorder, it can be classified as a mental impairment; hence a disability. The definition of mental health by the WHO (2010) is quite clear and it includes epilepsy. The WHO defines ‘mental health conditions’ as including schizophrenia, bipolar disorder, depression, epilepsy, alcohol and drug use disorders, child and adolescent mental health problems, and intellectual impairments. Epilepsy in Malawi, as well as at a global level, is therefore considered as a disability and this being the reason why persons with this condition were interviewed as part of this study.

## Methodology

### Research setting

This study was carried out in Traditional Authority (TA) Nankumba and TA Mponda in Mangochi District in southern Malawi. Mangochi lies on the shores of Lake Malawi and is situated 170 km north of Blantyre, the commercial capital of Malawi and about the same distance from Lilongwe, Malawi’s capital city. It has a population of 803 602 people; 387 072 are male whilst the rest are female (National Statistical Office 2008). The study was done in two fishing villages along Lake Malawi, three villages inland and around the headquarters of TA Nankumba, and one village at Mangochi District headquarters, which is part of TA Mponda. Fishing and farming are the major sources of income for the district.

### Research methods, research design and data collection

Whilst the wider study had both qualitative and quantitative components, this article only uses the qualitative data. The qualitative approach enabled us to explore people’s perceptions about the causes, treatment and prevention of various forms of disability and how having a disability impacted on households. The primary informants were persons with disabilities or their guardians, aged 18 years and above. For children with disabilities, interviews were instead conducted with their parents or guardians. A wide range of people with disabilities were interviewed, namely those with physical impairments, persons with albinism, persons with hearing impairments, persons with visual impairments, persons with intellectual disabilities and also with elderly men and women. Village headmen, community health workers and persons with disabilities assisted in identifying informants. A snowball method was also used to identify people with disabilities. This methodology involves informants referring the researcher to other informants, who are then contacted by the researcher. These informants in turn refer the researcher to yet other informants, and so forth (Noy 2008). At community level, there are not many health workers, hence these were identified by asking community leaders and other members of the community to help the research team identify these workers.

In total, 63 interviews were done with people with various forms of disabilities and 8 of these were with persons with epilepsy. The age range for these persons with epilepsy was between 4 years and 30 years: six persons were below 18 years and only two persons were above 18 years of age. All interviews were done with guardians of persons with epilepsy even for those who were above 18 years of age. This was mainly because they could not express themselves properly. A 30-year-old woman who had two children and was being taken care of by her grandmother. All the interviews were conducted by the researchers from Malawi and Norway.

Two group discussions were also conducted: one with mainly men with disabilities or parents of boys with disabilities (20 participants in this group), and another with women or parents of girls with disabilities (14 participants in this group). Interviews were also conducted with both professional and unskilled health workers and there were 21 such interviews: 4 were with traditional healers; 6 with health surveillance assistants (HSAs); 3 with traditional birth attendants; 2 with community health volunteers; 2 with medical doctors and the rest with orthopaedic technicians. HSAs are the lowest cadre in the Ministry of Health, based at community level, and they have a catchment area of about 1000 people each (Ministry of Health 2011). The interviews with health workers were mainly from what Kleinmann (1980) calls the ‘folk’ and ‘professional sectors’. The professional sector is composed of the organised healing professions and in this context provided by the Ministry of Health. The folk sector is the non-professional, non-bureaucratic, specialist sector, encompassing both sacred and secular healers, such as traditional healers, shamans and folk psychotherapists (Kleinmann 1980). These interviews and group discussions were conducted in March 2009. Persons with disabilities were asked about the type of disability they suffered from, their perceptions about the causes of their disability, where they sought treatment and the impact of having a person with a disability within the household. These same issues were explored with persons with epilepsy.

### Study limitations

The major limitation of this study is that it is based on only eight cases of persons with epilepsy, but this is not unusual in qualitative studies. The study nevertheless brings out the health seeking behaviour of persons with epilepsy.

## Ethical considerations

The study received ethical approval from Malawi’s National Health Sciences Research Committee (NHSRC) and the Committee for Medical and Health Research Ethics in Norway. The NHSRC is an institutional review board whose secretariat is in the Ministry of Health and draws membership from various institutions. All participants in this study were informed about its objectives and informed consent was obtained prior to the interviews. Participants were assured of the confidentiality of the information they shared with the research team. Their participation was voluntary and they were also at liberty to withdraw from the interview at any time they felt so. All the names used in this article are fictitious in order to protect the identity of informants.

## Data analysis

In depth interviews with persons with disabilities were recorded and translated into English. These interviews were typed in Microsoft Word. The eight interviews with persons with epilepsy, or their caretakers, were analysed using content analysis. They were read several times and recurring themes were identified. For purposes of this article, the analysis focused on people’s perceptions about the causes of epilepsy, how persons with epilepsy seek health care, barriers to seeking care for this condition, and the impact of having a person with epilepsy in the household. The analysis of the data was done by the researchers themselves.

## Results

### Perceptions about the causes of illness

In the Mangochi area where this study was conducted, epilepsy is commonly known as *khunyu* in the local language. The biomedical causes of epilepsy have been explained earlier but they are not universally accepted as communities have their own perceptions about the causes of this condition. In the current study, some guardians did not even know the cause of epilepsy. Participant (P) 1, a mother of an 11-year-old boy with epilepsy, explained how her child developed epilepsy and how the family sought treatment:

The epilepsy started in 2003. One day he fainted [*kugwa khunyu*] 10 times. She took him to the hospital because she thought it was malaria. The doctor at Mangochi District Hospital explained that the boy had epilepsy but the hospital did not have medication for epilepsy at the time so he advised them to go to a traditional healer. The traditional healer also did not have a cure for epilepsy and he did not give any explanation on what caused it. He only explained that it was the will of God and there was nothing he could do about it. They went back to the hospital after three years and the hospital has been giving them some medicines for epilepsy. At least the medication has helped because now he can go out and play with friends, which he could not do before. However, he still faints [*amagwa*] sometimes and he still cannot talk. (Reported speech of an interview with P1)

P1 did not know that her child suffered from epilepsy and it was only the doctor who told her that it was epilepsy. She therefore lacked knowledge about the disease. In this case even the traditional healer said that the disease that P1’s child suffered from, was the *will of God*. The fact that this traditional healer attributed P1’s son’s illness to the *will of God* does not imply that this is the only interpretation that people in Mangochi use to explain the cause of epilepsy. Another traditional healer (P2) said that epilepsy can also be caused by witchcraft; witches tend to put faeces of a mouse on the stomach of the new baby which is mixed with herbs. Another healer in Mangochi, P3, attributed epilepsy to the presence of too much foam in the stomach and that the onset of diarrhoea is a sign that the person with epilepsy is getting better.

Apart from witchcraft, *the will of God* and the presence of too much foam in the stomach, epilepsy was also said to be caused after experiencing serious illness. In the majority of cases interviewed in this study, the genesis of disability (including epilepsy) was preceded by serious illness and in most cases informants cited malaria as a cause of disability, as the case of P4’s son below demonstrates:

P4’s son was okay at birth. He was born in 2004. He had very high fever some times and then he fell unconscious. His mother remembers that during one day in 2008 he fell unconscious 30 times and they did not know what caused the illness. When they went to the hospital, they were told that the child had malaria and he was given treatment but the situation never changed. After some time they were told by the doctor that it was *khunyu* [epilepsy]. (Reported speech of an interview with P4)

As was the case with P4’s son, it was reported that most of the other patients with epilepsy first suffered a serious illness such as malaria. Even after being given treatment, the fits or seizures did not stop.

### Seeking treatment for epilepsy

Persons with epilepsy or their guardians were asked about where they had sought treatment for their condition. Both modern health facilities and traditional healers were mentioned, as can be seen above in the discussion with P1, whose 11-year-old son had epilepsy. Whilst people may not want to go and consult traditional healers, the general lack of medicines tends to force people to seek treatment from traditional healers, as was the case with P1’s son. It is also striking that it was a health worker who advised P1 to seek traditional medicine for her son. The treatment that persons with epilepsy get from the health facilities is quite effective as it reduces the fits but it does not take them away completely. Several informants said that if they forgot to administer the medicine then the patients would get fits right away. The traditional healer who said that epilepsy can be caused by witchcraft, explained that he could easily cure such type of epilepsy by rubbing traditional herbs on the person with epilepsy, and that this was effective so long as treatment is sought before the age of two. He did not explain why this was the case. Purging is also an important part of the process of healing, as was also mentioned by a traditional healer. This was especially the case as it was perceived that epilepsy can also be as a result of too much foam in the stomach.

### Barriers to seeking therapy for epilepsy

This study also found quite a number of barriers to accessing therapy for epilepsy. The case of P1’s son illustrates that one of the barriers to seeking care for epilepsy is the general lack of medication in modern health facilities. The doctor told P1 that there were no medicines in the facility for epilepsy; hence she had to go to a traditional healer who could not help either. This was also highlighted by a number of informants in this study. Distance to health facilities is also a major determinant of therapy seeking for persons with epilepsy and their guardians, as can be seen from a summary of discussions with P5, the father of a 23-year-old man with epilepsy:

The family of P5 gets the medication they need for free from the health facility. The only problem is that they have to travel as far as Mangochi District Hospital every Wednesday to get it. The father has tried asking if it would be possible to get it from the nearby clinic. It worked once when they got it from the neighbouring clinic but after this they were told that they had to go back to the hospital from that time on. This is a considerable strain both on time and the economy. (Reported speech of an interview with P5)

P5 resides in Chembe Village, ±50 km away from Mangochi District Hospital where they have to get the treatment for epilepsy. The medicines for epilepsy are not available at the nearby clinic. Arrangements have been made to have these medicines at the clinic, but this has either been discontinued or the supply of medicines to health facilities has been erratic. Whilst health services are provided free of charge in Malawi, including accessing treatment for epilepsy, the cost of transport affects access to treatment, as is the case with P5’s family. The issue of transport was also illustrated by P6, a woman with a granddaughter with epilepsy in Msaka Village in the same district:

The birth of P6’s granddaughter was normal, but she suffered from epilepsy already as a child and she still does. She gets medication from Monkey Bay Hospital. The medication reduces the fits but does not take them away completely. The problem with getting the medication is that they have to go to Monkey Bay to collect it. This is a long way to go and they do not manage to get there regularly. But they know a nurse who works there and who comes from their area and she sometimes brings the medication to them. (Reported speech of an interview with P6)

Persons with epilepsy need to take medication regularly in order to prevent seizures. Because of distance, as is the case with P6’s granddaughter and other persons with epilepsy, this is not always possible; hence there is failure to adhere to treatment which results in the patients having frequent seizures. The presence of a health worker in the facility who is a relative or someone from home helps in terms of ensuring that medication is available. In addition to distance to health facilities, P1 also reported that:

There is congestion at the hospital. As such, when the child has fainted or when he is in serious condition, they still have to be in the queue until it is their turn to be assisted. Sometimes there is a shortage of drugs, although most of the time the drugs are available. Sometimes they have to hire a bicycle and pay $0.17 because the mother cannot manage to carry him on her back and take him to the hospital. (Reported speech of an interview with P1)

Congestion in health facilities, as mentioned by P1, is one of the problems that she experienced in seeking care for treatment of epilepsy. This means that the patients and their guardians have to wait for a long time before being seen by a health worker at the health facility. What we see, therefore, is that from the eight cases examined in this article, both modern and traditional medicine are used in the treatment of epilepsy and, because they have experienced the failure of traditional medicine, most of the patients or their guardians are taking modern medicine. However, the facilities are situated quite far away, and in some cases transport is not available for the patients and their guardians to go there.

### Impacts of epilepsy on the household

During interviews, informants were further asked about how having a patient with epilepsy affects the family. In a discussion with P7, she summarised the daily life of her son (8 years) who has epilepsy as follows:

There is nothing that he does. When he wakes up he sits down the whole day. In terms of hygiene, toilet, dressing, et cetera, the mother does everything for him. She feeds him, washes his clothes and clothes him. She says that his right side is too weak to do anything. She also reports that he does not participate in any social activities and does not go to school because she feels he can’t do anything such as write or talk and also it would be difficult in terms of transportation to school because he can’t go there on his own. (Reported speech of an interview with P7)

P5’s son was a special case as he was also paralysed on the right side of his body and he could not talk. His mother summarised quite well the lives of some people who have epilepsy. Everything has to be done for them, their participation in social activities is restricted and the chances of going to school are almost non-existent as they would always need to be escorted. Like some other patients with epilepsy, P8’s son started school and was doing quite well but when the seizures were severe they affected him and he eventually dropped out of school. P8 reported that when their son has long seizure episodes, he becomes restless and wanders around and behaves like a ‘madman’, hence his parents have to be with him constantly for at least two days after a seizure. In some cases, some of the children with epilepsy have not been to school because their parents think that there is no one to look after them at school if they have an epileptic episode. There are other patients, however, who are able to do some things on their own, as was the case with P5’s son, who was able to do some simple things around the house including washing and dressing himself and did not need to be reminded.

The presence of a person with epilepsy in the home also affects household productivity, for example:

P7’s mother said that when her son’s situation was very critical, not a single member of the household could go to the garden or do any *ganyu* [piecework]. They stayed at home and looked after the boy. She said that she could not go out to work or look for work since she always had to stay at home and look after her son. (Reported speech of an interview with P7)

In another such case, P9, the father of a son with epilepsy, for example, absented himself from work to care for his child, especially when the condition was severe. In communities where this study was done, poverty is widespread, as is the case with the rest of the country. Whilst household members can absent themselves from work, in some cases it is not possible to do this. Many children with disabilities, including those with epilepsy, are in some cases left alone because there is no one else in the household to care for them.

In an interview with P10 in Mangochi, she said that her child did not walk and had epilepsy. She was left alone whilst she (the mother) went to the field:

The girl was left alone, locked up in the house from 06:00 when the mother went to the fields until around 09:00. If the mother found food she came back home, otherwise not. The younger sister was left outside the house to play with the other children. The mother said there was no one they could ask for help because there was no one who would have agreed with their heart. She said the girl did not cry when she was alone. The mother worried about her when she had to leave her. (Reported speech of an interview with P10)

P10 had no choice but to go and work and make ends meet for the family.

Some of the parents used to run small-scale businesses but they ended up using their capital whilst seeking treatment:

On how the child’s illness has affected her life, P4 said that she used to run businesses such as selling rice, tomatoes and groundnuts. She bought rice in bulk from Zomba whilst the tomatoes were bought in Dedza. She bought groundnuts in Cape Maclear. She used to sell them at the local market in Chembe village every day. She also used to take care of the home, washing, *kuzira* (smearing the floor with mud), cooking and ironing. Her husband used to construct kitchens and toilets amongst other duties. When their child suffered from epilepsy, they used all the money that they had for running their business seeking care for the child. Because of the child’s sickness, they were poor at the time of the study – they had no food and no clothes. In addition, the child was sick quite often and they could not adequately take care of him and the rest of the family. Sometimes they could do *ganyu* [piecework] but the money was spent on seeking care for the child. The *ganyu* they did included working in other people’s fields and fetching firewood and selling it. There was no one who helped in caring for the child. Her mother-in-law had goats but she sold all of them to help the family when the child was admitted. She used to help by giving the family money and food but at the time of the fieldwork all the goats had been sold and she could not help them anymore. (Reported speech of an interview with P4)

P10’s business closed down, as she used the money whilst seeking care from traditional healers and modern health facilities. There were also others whose businesses closed down because they spent all the money on seeking care. Whilst some would like to engage in business, they fail to do that because they always have to be with their child with epilepsy. P10 therefore suggested that in order to address the problems she was facing, she needed a loan to establish an income-generating activity.

In addition to the problems that the households with persons with epilepsy experience, people with epilepsy themselves also experience problems, especially discrimination, as narrated by P1:

The child is chased from other homes because other households feel like the child wants to beg some food, so they do not even offer him any food. Some people also say that he is ugly and stupid because he has saliva coming out of his mouth. As such, the child is discriminated against. He is even chased by friends when he tries to play with them because he cannot talk so he cannot communicate well with his friends. When this happens, he cries and the mother has to take him away from his friends. (Reported speech of an interview with P1)

This case demonstrates that there is stigma and discrimination associated with epilepsy. This section has demonstrated that having a person with epilepsy within the household has wide ramifications especially if the disorder is not controlled. Improving access to treatment can minimise these impacts of epilepsy on households.

## Discussion

In terms of knowledge about epilepsy in the study area, there were a number of informants who reported that they did not know about epilepsy hence they were just informed by the doctors after diagnosis that their children had this condition. It is not only community members who may not know about epilepsy but health workers themselves as well. P4’s case also demonstrates that there can be misdiagnosis at the health facility: at first the health workers told parents that the child had malaria and then later they said that it was epilepsy. In other countries such as Tanzania, researchers have also found that late or misdiagnosis is a major challenge in the management of epilepsy and that this delays the onset of treatment until the time it is properly diagnosed (Mushi *et al.* 2012).

In the study, some caretakers said that their children suffered from epilepsy after suffering from a serious illness for a long time. The Sue Ryder Foundation is involved in the provision of treatment to persons with epilepsy in southern Malawi and reports that 39% of the people with epilepsy cared for by their nurses had cerebral malaria in early childhood (Sue Ryder Foundation 2009). It was a traditional healer who said that the disease can also be caused by witchcraft. A more recent study done by the Federation of Disability Organisations in Malawi also found that epilepsy is believed to be linked to witchcraft and spirits (Amos & Wapling 2010). A number of studies have been done in other African countries which have found that witchcraft is believed to be a cause of epilepsy (Mushi *et al*. 2012). Baskind and Birbeck (2005) found that most traditional healers in Zambia believed that witchcraft was to some extent responsible for seizures. In some parts of Cameroon these beliefs were widespread and 50% of the respondents actually attributed epilepsy to witchcraft (Njamnshi *et al.* 2009). The perception is that epilepsy caused by witchcraft can only be cured by traditional healers.

Another traditional healer reported that epilepsy is also caused by too much foam in the stomach. This finding is similar to that of Nkwi and Ndoko (1989), who did their study amongst the Bamileke of Cameroon, amongst whom epilepsy is perceived as a saturation of foam in the stomach which goes to the head and makes the eyes turn and the victim fall. Hence, treatment requires that the process of purging should be initiated with traditional medicine to expel existing foam from the stomach (Nkwi & Ndonko 1989). It was also observed that a traditional healer attributed epilepsy to the *will of God*. In this case, the onset of epilepsy is not due to witchcraft and other supernatural forces but that the disease just comes, meaning that it is naturally caused. The onset of epilepsy in this case is ‘merely part of the existential reality of the world’ (Friedson 1996) or ‘part of the expected order’. Ngubane (1977) calls such diseases ‘natural illnesses’, because they just happen and do not result from personal malice or the fault of the patient. Some anthropologists have called such diseases *illnesses of God*: not that it is God who causes them but that they just happen (Feierman 1981). The traditional healer attributed P1’s son’s illness to the *will of God*, implying that it was a natural illness. Other studies (e.g. Mushi *et al*. 2012) have further attributed epilepsy to a disease that is inherited, but none of informants in the current study mentioned this. Other causes of epilepsy that have been identified in Malawi include inheritance, worries and brain tumours, amongst others (Chilopora *et al*. 2001). In Swaziland, Reis (1994) describes a belief in which a snake in the belly causes convulsions by raising itself in the body.

In terms of treatment, a traditional healer mentioned that initiating purging is an important component of healing. Other studies have also found that in some African societies, including Malawi, epilepsy is believed to be caused by something like an insect that moves around in the stomach and that traditional healers use a concoction made from roots to induce purging and vomiting (Watts 1989; Whiteley 2005). As Diop *et al*. (2003) report, it is evident that the social cultural perception of the causation of disease is one of the major determinants of therapy seeking for patients with epilepsy (Diop *et al*. 2003). The results from the current study and other studies done in Africa show that there is double utilisation of western and traditional medicines by most people suffering from epilepsy. For example, Mushi *et al*. (2012) in Tanzania, also found that both modern and traditional forms of treatment are used during episodes of epilepsy and that most people started by taking children with epilepsy to the health centre and when it persisted they went to faith or traditional healers. The majority of persons with epilepsy tend to seek professional care very late.

This study also found quite a number of barriers to accessing therapy for epilepsy. The case of P1’s son illustrates that one of the major barriers to seeking care for epilepsy is the general lack of medication in modern health facilities. The doctor told P1 that there were no medicines in the facility for epilepsy; hence she had to go to a traditional healer, who could not help either. In a study done in the Burns Unit at Queen Elizabeth Central Hospital in Blantyre, southern Malawi, which caters for about 4 million people, it was found that 4 out of 12 patients examined had epilepsy and they sustained burns during epileptic fits near a paraffin lamp or an open fire. None of the patients with epilepsy were on any anticonvulsant medication owing to lack of drugs (Virich & Lavy 2006). In some cultures, burns from a person with epilepsy falling into a fire are looked at differently, for example, in Zimbabwe, Mugumbate and Mushonga (2013) found that if persons with epilepsy are burnt in fires, they will not respond to treatment.

Congestion in health facilities, as mentioned by P1, is one of the problems that is experienced in seeking care for treatment of epilepsy. This makes patients and their guardians wait for a long time at the health facility before being seen by a health worker. In a study done in Lusaka, Zambia, Frankenberg and Leeson (1976) found that some traditional healers are consulted because, amongst other factors, their services were available without queuing. The experience of long queues can make some people consult traditional healers. It is evident that the therapy-seeking process for epilepsy is not a random process but an ordered path of choices responding to negative feedback. As Crandon-Malamud (1991) argues, boundaries between different therapeutic options are not rigid, as people move from one form of therapy to another.

This study has also shown that access to education is a problem for children with epilepsy as they would always need to be escorted. Some patients with epilepsy, such as P8’s son, start school and do quite well, but in his case, when the seizures were severe, they affected him and he eventually dropped out of school. A study conducted by the Federation of Disability Organisations in Malawi actually shows that 69% of respondents with epilepsy have never been to school. Of the 31% that attended, just 0.3% went beyond secondary school level. This decreases their chances of employment, hence increasing, their likelihood of being poor (see Amos & Wapling 2010). Mushi *et al*. (2012) in Tanzania and Komolafe *et al*. (2012) in Nigeria also found that children with epilepsy did not attend school regularly because of on-going seizures, or they had fewer years of formal education.

As has been demonstrated in this study, some parents could not work just because they had to care for the child with epilepsy especially when the condition was severe. Priority is therefore given to the care of children with disabilities, as was also seen in a study done in Tanzania, where Mushi *et al*. (2012) found that the carer’s ability to work and provide for the family, is impaired. Poverty is widespread in the communities where the current study was done, as is the case with the rest of the country. A recent national survey found that 50.7% of households were living below the poverty line (National Statistical Office 2012). In contexts where poverty is widespread, medicines for epilepsy are unavailable and seizures are common, the impact at household level can be quite significant as households can be pushed into poverty, as demonstrated above.

Epilepsy also carries with it an enormous amount of stigma and discrimination, as has been demonstrated in this study. This is mainly because of the prevailing misconceptions about how epilepsy is caused. In many communities, children will not play with fellow children with epilepsy. In Cameroon, Njamnshi *et al*. (2009) report that nearly a fifth of their respondents said that they would object to their children associating with persons with epilepsy. Nkwi and Ndonko (1989) and Njamnshi *et al.* (2009) have even found that epilepsy can become a social stigma as people will refuse to marry into a family where there is a case. Whilst some friendship can be maintained, in some cases it is disrupted and children with epilepsy are isolated (Mushi *et al*. 2012). Basking and Birbeck (2005) explain that people actually believe that seizures are contagious and are spread through saliva, urine and faeces, and the fear of contagion causes stigma.

## Conclusion

This study, as is the case with other studies that have been done in sub-Saharan Africa, generally demonstrates that misperceptions about the causes of epilepsy exist in Malawi. The prevailing cultural beliefs influence the way persons with epilepsy and their families will seek health care. As has been demonstrated in this study, beliefs about witchcraft as a cause of epilepsy are widespread, and since it is believed that diseases caused by witchcraft cannot be cured with western medicines, persons with epilepsy will seek care from traditional healers as well. Other factors such as widespread poverty, misdiagnosis by health workers, the lack of medicines for epilepsy in the health centres and distance to health facilities tend to hinder access to effective epilepsy treatment.

As a result of these factors, a significant proportion of persons with epilepsy in resource-poor countries such as Malawi are not on treatment. The lack of medicines makes persons with epilepsy suffer from frequent seizures, which negatively impacts on households. Despite prevailing barriers to accessing treatment for epilepsy, an earlier study conducted in Malawi demonstrated that it is possible to address existing barriers. Watts, working at a hospital in northern Malawi and using a combination of public education, simple treatments, regular reviews and ensuring an adequate supply of free drugs, developed a community-based epilepsy treatment programme from nothing to treating 461 patients over the course of two years (Watts 1989). Such a community-based approach can be used to close the treatment gap for epilepsy as is being advocated by the Global Campaign against Epilepsy.
